# A higher non‐HDL‐C/HDL‐C ratio was associated with an increased risk of progression of nonculprit coronary lesion in patients with acute coronary syndrome undergoing percutaneous coronary intervention

**DOI:** 10.1002/clc.24243

**Published:** 2024-02-25

**Authors:** Jiamin Liu, Li Zhao, Yazhou Zhang, Lili Wang, Qianqian Feng, Jing Cui, Wenhong Zhang, Jianyong Zheng, Dan Wang, Fengjiao Zhao, Jiangchun He, Yu Chen

**Affiliations:** ^1^ The Second School of Clinical Medicine Southern Medical University Guangzhou China; ^2^ Department of Cardiology Sixth Medical Center of PLA General Hospital Beijing China; ^3^ The Fourth Affiliated Hospital of Inner Mongolia Medical University Baotou China; ^4^ Fengning Manchu Autonomous County Hospital Chengde China

**Keywords:** acute coronary syndrome, nonculprit lesion progression, non‐HDL‐C/HDL‐C ratio, percutaneous coronary intervention

## Abstract

**Background:**

The ratio of nonhigh‐density lipoprotein cholesterol (non‐HDL‐C) to high‐density lipoprotein cholesterol (HDL‐C) has been shown associated with various metabolic diseases and atherosclerosis in primary prevention. However, there is limited evidence on the relationship between the non‐HDL‐C/HDL‐C ratio and progression of nonculprit coronary lesion (NCCL) after percutaneous coronary intervention (PCI).

**Hypothesis:**

Our study aimed to investigate the potential association between the non‐HDL‐C/HDL‐C ratio and NCCL progression in patients with acute coronary syndrome (ACS) undergoing PCI.

**Methods:**

We conducted a retrospective analysis of ACS patients who underwent coronary angiography twice at a single center from 2016 to 2022. Lipid measurements, demographic, clinical, and other laboratory data were collected from electronic medical records. NCCLs were evaluated using quantitative coronary angiography. The primary outcome was the progression of NCCL. Patients were categorized based on NCCL progression and tertiles of the non‐HDL‐C/HDL‐C ratio. Associations were analyzed using univariate and multivariate logistic regression analysis.

**Results:**

The study included 329 ACS patients who underwent PCI, with a median follow‐up angiography of 1.09 years. We found NCCL progression in 95 (28.9%) patients with acceptable low‐density lipoprotein cholesterol control (median: 1.81 mmol/L). Patients in the top tertile of the non‐HDL‐C/HDL‐C ratio had a higher risk of NCCL progression. After adjusting for potential confounding factors, the non‐HDL‐C/HDL‐C ratio remained a significant predictor for NCCL progression (adjusted odds ratio: 1.45; 95% confidence interval: 1.14–1.86; *p* < 0.05).

**Conclusions:**

The non‐HDL‐C/HDL‐C ratio predicts NCCL progression in ACS patients following PCI, providing a valuable tool for risk assessment and enhancing secondary prevention of atherosclerotic cardiovascular disease.

## 1 INTRODUCTION

Percutaneous coronary intervention (PCI) combined with standard drug treatment has proven effective in reducing mortality in patients with acute coronary syndrome (ACS). However, even after successful PCI for culprit vessels, there are still risk factors of recurrent major adverse cardiovascular events (MACE). In addition to in‐stent restenosis, over 50% of these recurring incidents can be attributed to the progression of nonculprit coronary lesions (NCCLs).^1^, ^2^, ^3^ To mitigate cardiovascular risk, international guidelines underline the management of low‐density lipoprotein cholesterol (LDL‐C) as a fundamental strategy.^4^, ^5^, ^6^ Despite efforts to maintain LDL‐C levels within recommended targets through medication, there remains a significant residual risk of atherosclerosis.^7^ This residual risk includes various factors, such as inflammation, hypertension, diabetes, and other metabolic abnormalities. Among these factors, residual cholesterol emerges as a crucial target requiring further exploration.

Nonhigh‐density lipoprotein cholesterol (non‐HDL‐C) is recommended for cardiovascular risk estimation in individuals with elevated levels of triglyceride (TG), diabetes mellitus, obesity, or extremely low levels of LDL‐C, according to current US, Canadian, and European guidelines.^4^, ^5^, ^6^ It represents the total content of atherogenic lipoproteins containing apolipoprotein B (apoB). On the other hand, several observational studies have shown an inverse relationship between plasma HDL‐C and the risk of atherosclerotic cardiovascular disease (ASCVD). Previous studies have also indicated a connection between the non‐HDL‐C to HDL‐C ratio and arterial stiffness,^8^ nonalcoholic fatty liver diseases,^9^ chronic kidney disease,^10^ diabetes,^11^, ^12^ metabolic syndrome,^13^, ^14^ and insulin resistance.^13^, ^14^ These findings suggest that the non‐HDL‐C/HDL‐C ratio may serve as a superior predictor compared to non‐HDL‐C or HDL‐C alone. However, there is limited research on the association between the non‐HDL‐C/HDL‐C ratio and the progression of coronary atherosclerosis, especially in NCCL of ACS patients following PCI.

In a retrospective cohort of ACS patients who have undergone PCI, we aimed to explore the potential predictors of NCCL progression and to assess if the non‐HDL‐C/HDL‐C ratio could be utilized as a stronger independent predictor of NCCL progression compared to LDL‐C under contemporary guideline‐directed medical therapy.

## 2 PATIENTS AND METHODS

### 2.1 Study design and population

This was a single‐center, retrospective cohort study. A total of 1042 patients underwent consecutive coronary angiography (CAG) procedures between January 2016 and December 2022 at the Sixth Medical Center of PLA General Hospital, Beijing, China.

The inclusion criteria for this study were as follows: (1) ACS patients who received PCI for culprit lesions during the initial CAG or previous CAG; (2) there must be at least one major vessel or branch vessel with a diameter of no less than 2 mm present without treatment during the initial angiography; and (3) the time between the two CAGs needed to be at least 3 months, excluding those who underwent staging procedures in the short term.

The exclusion criteria involved: (1) a history of coronary artery bypass grafting before the initial CAG; (2) severe cardiac insufficiency, defined as a left ventricular ejection fraction less than 40%; (3) severe liver and/or renal insufficiency; (4) malignant tumors, acute infections, or immune system diseases; and (5) incomplete clinical or coronary angiography data.

After excluding 649 patients who had a second coronary angiography within 3 months, and 64 patients who met other exclusion criteria, 329 patients were included in the current analysis (the study population flowchart can be found in Supporting Information: Figure 1).

This study adhered to the Declaration of Helsinki and was approved by the Ethics Review Committee of the Sixth Medical Center of PLA General Hospital, Beijing, China. Written informed consent was waivered by the Ethics Review Committee as this was a retrospective study.

### 2.2 Data collection

The clinical data of the patients were collected and recorded through the electronic medical information management system at the Sixth Medical Center of PLA General Hospital in Beijing, China. Basic demographic information, such as sex, age, height, weight, and blood pressure upon admission, was extracted from the electronic nursing records. Prior medical history of the patients was obtained from the admission records, while information regarding medication usage was gathered from the discharge records following the initial CAG.

The present study focused on the routine blood lipid levels of patients before undergoing CAG, including LDL‐C, HDL‐C, total cholesterol (TC), and TG. To acquire this data, we accessed the Laboratory Information System (LIS) and retrieved the most recent test results before the CAG procedure. Blood lipids were measured using automatic biochemical analyzers (AU5800 or AU5821; Beckman Coulter) in our hospital laboratory. The concentration of LDL‐C was determined through a direct method with surfactant removal technique, while the concentration of HDL‐C was determined using a direct method with catalase clearance technique. TC was measured by enzymatic methods, and TG was measured using the GPO‐POD method. Non‐HDL‐C was calculated using the equation: Non‐HDL‐C = TC − HDL‐C.

The coronary angiograms at baseline and follow‐up were analyzed using a validated quantitative coronary angiography system (QCA Quantcor; Siemens). This system allowed us to measure parameters such as minimal lumen diameter, reference vessel diameter, diameter stenosis, and the length of both the culprit and nonculprit lesions.

### 2.3 Definitions

An NCCL was defined as a de novo stenotic lesion that did not result in ischemic symptoms or show positive results on functional ischemic tests. In instances where multiple NCCLs were present, the primary lesion for each patient was determined based on the largest increase in diameter stenosis observed in the follow‐up CAG. Patients were categorized into the angiographic NCCL progression group if they met any of the following criteria: a decrease in diameter of ≥10% in a pre‐existing stenosis that was initially ≥50%; a decrease in diameter of ≥30% in a pre‐existing stenosis that was initially <50%; development of a new stenosis with a diameter decrease of ≥30% in a segment that was normal in the initial diagnostic CAG; or progression of any lesion to total occlusion in the subsequent follow‐up CAG.^15^ Hypertension was defined as systolic blood pressure exceeding 140 mmHg, diastolic blood pressure exceeding 90 mmHg, or a self‐reported history of hypertension and use of antihypertensive medications. Diabetes mellitus was defined as a medical history of the disease, use of antidiabetic drugs, or blood glucose levels exceeding the criteria set by the American Diabetes Association.^16^

### 2.4 Statistical analysis

Statistical analysis was performed using SPSS, version 25 (SPSS Inc.) and R software version 4.3.0 (R Core Team). The subjects were classified based on the observed progression of NCCL during the follow‐up and the tertiles of the baseline non‐HDL‐C/HDL‐C ratio. Continuous variables were presented as the mean ± standard deviation (SD) or median with the 25th and 75th percentiles, as deemed appropriate. Gaussian distribution variables were analyzed using the *t*‐test or ANOVA test, while the Mann–Whitney *U* test or Kruskal–Wallis H test was used for non‐Gaussian distribution variables. Categorical variables were expressed as counts and percentages, and comparison was made using the *χ* ^2^ test or Fisher's exact test. Univariate logistic regression analysis was performed to identify potential risk factors for NCCL progression. Additionally, multivariate logistic regression was conducted to determine whether the non‐HDL‐C/HDL‐C ratio and other isolated lipid parameters could independently predict the progression of NCCL.

## 3 RESULTS

A total of 329 patients were included in the final analysis, with a mean age of 61.9 ± 10.6 years. Among these individuals, 259 (78.7%) cases were male. During a median follow‐up period of 1.09 years (interquartile range: 0.80–1.93 years), 95 (28.9%) patients experienced NCCL progression (Table 1). Baseline demographic and clinical characteristics were compared between the patients who experienced NCCL progression and those who did not in Table 1. There were no statistically significant differences observed in age, sex distribution, body mass index (BMI), admission blood pressure and heart rate, history of hypertension and diabetes, smoking habits, previous PCI history, or history of myocardial infarction between the group experiencing NCCL progression and the nonprogression group. Additionally, there were no significant differences in terms of the baseline diagnosis of acute myocardial infarction or the number of PCI‐treated lesions between the two groups. Following the baseline PCI procedure, all patients received pharmacologic therapy as determined by the treating physician.

**Table 1:** Baseline characteristics of the study population with and without NCCL progression during follow‐up.

		Total (*n* = 329)		No NCCL progression (*n* = 234)	NCCL progression (*n* = 95)	*p*
Age (years)		61.9 ± 10.6		61.6 ± 10.7	62.8 ± 10.3	0.35
Male, *n* (%)		259 (78.7)		182 (77.8)	77 (81.1)	0.51
BMI (kg/m^2^)		25.6 ± 3.20		25.5 ± 3.23	25.7 ± 3.12	0.62
SBP (mmHg)		136.1 ± 19.7		135.7 ± 19.5	137.0 ± 20.1	0.59
DBP (mmHg)		79.6 ± 12.8		80.0 ± 11.5	78.5 ± 15.6	0.33
Heart rate (beats/min)		75.6 ± 12.5		76.1 ± 12.4	74.4 ± 12.5	0.26
Hypertension, *n* (%)		229 (69.6)		159 (68.0)	70 (73.7)	0.31
Diabetes mellitus, *n* (%)		152 (46.2)		109 (46.6)	43 (45.3)	0.83
Smoking, *n* (%)		193 (58.7)		138 (59.0)	55 (57.9)	0.86
Previous MI, *n* (%)		49 (14.9)		37 (15.8)	12 (12.6)	0.46
Previous PCI, *n* (%)		124 (37.7)		91 (38.9)	33 (34.7)	0.48
AMI, *n* (%)		60 (18.2)		37 (15.8)	23 (24.2)	0.07
No. of PCI‐treated lesion, *n*		1.8 ± 0.9		1.8 ± 0.9	1.7 ± 1.0	0.60
Lipid lowering drugs, *n* (%)		328 (99.7)		233 (99.6)	95 (100.0)	1
Antiplatelet drugs, *n* (%)		329 (100.0)		234 (100.0)	95 (100.0)	1
ACEI/ARB, *n* (%)		175 (53.2)		125 (53.4)	50 (52.6)	0.90
β‐blocker, *n* (%)		247 (75.1)		177 (75.6)	70 (73.7)	0.71
CCB, *n* (%)		98 (29.8)		70 (29.9)	28 (29.5)	0.94
Follow‐up period (years)		1.09 (0.80–1.93)		1.05 (0.84–1.75)	1.13 (0.65–2.19)	0.47

*Note*: Values are mean ± SD, median (interquartile range), or *n* (%).

Abbreviations: ACEI, angiotensin‐converting enzyme inhibitor; AMI, acute myocardial infarction; ARB, angiotensin II receptor blocker; BMI, body mass index; CCB, calcium channel blocker; DBP, diastolic blood pressure; MI, myocardial infarction; NCCL, nonculprit coronary lesion; PCI, percutaneous coronary intervention; SBP, systolic blood pressure.

Patients with NCCL progression had a higher non‐HDL‐C/HDL‐C ratio at baseline than those without progression (*p* = 0.011, Table 2). At baseline, there was no significant difference in isolated lipid parameters such as LDL‐C, TC, and TG between the two groups. However, HDL‐C levels were consistently lower in patients with NCCL progression than in those without NCCL progression (all *p* < 0.05). Additionally, all patients showed improved control of atherogenic cholesterol bound to LDL and remnants during follow‐up (Table 2).

**Table 2:** Blood lipids at baseline and follow‐up of patients with and without NCCL progression.

	Total (*n* = 329)	No NCCL progression (*n* = 234)	NCCL progression (*n* = 95)	*p*
TC (mmol/L)				
Baseline	4.18 (3.37–5.00)	4.08 (3.35–4.83)	4.33 (3.41–5.21)	0.30
Follow‐up	3.41 (2.94–4.01)	3.45 (2.94–4.05)	3.36 (2.96–3.93)	0.61
Change	−0.50 (−1.52–0.04)	−0.46 (−1.33–0.03)	−0.84 (−1.88–0.03)	0.12
TG (mmol/L)				
Baseline	1.54 (1.15–2.25)	1.52 (1.15–2.28)	1.58 (1.15–2.18)	0.68
Follow‐up	1.33 (0.97–1.85)	1.35 (0.97–1.87)	1.23 (0.94–1.81)	0.34
Change	−0.17 (−0.71–0.16)	−0.12 (−0.59–0.23)	−0.27 (−0.89–0.07)	0.068
HDL‐C (mmol/L)				
Baseline	1.10 (0.93–1.29)	1.12 (0.94–1.32)	1.08 (0.91–1.23)	0.047
Follow‐up	1.07 (0.94–1.22)	1.10 (0.97–1.26)	1.03 (0.91–1.17)	0.011
Change	−0.01 (−0.14–0.10)	−0.02 (−0.14–0.10)	0.00 (−0.14–0.09)	0.83
LDL‐C (mmol/L)				
Baseline	2.30 (1.72–2.99)	2.25 (1.72–2.86)	2.40 (1.71–3.25)	0.20
Follow‐up	1.81 (1.48–2.24)	1.81 (1.48–2.17)	1.76 (1.48–2.29)	0.62
Change	−0.39 (−1.12–0.03)	−0.38 (−1.01–0.03)	−0.48 (−1.36–0.01)	0.31
Non‐HDL‐C (mmol/L)				
Baseline	3.03 (2.27–3.79)	2.95 (2.25–3.63)	3.15 (2.35–4.07)	0.11
Follow‐up	2.28 (1.88–2.86)	2.29 (1.81–2.87)	2.22 (1.94–2.83)	0.96
Change	−0.34 (−1.06–0.28)	−0.27 (−0.96–0.36)	−0.54 (−1.36–0.14)	0.020
Non‐HDL‐C/HDL‐C				
Baseline	2.70 (2.10–3.42)	2.66 (2.00–3.27)	2.88 (2.23–3.79)	0.011
Follow‐up	2.16 (1.64–2.66)	2.13 (1.62–2.60)	2.23 (1.81–2.86)	0.16

*Note*: Values are median (interquartile range). Change in blood lipids = follow‐up lipids − baseline lipids.

Abbreviations: HDL‐C, high‐density lipoprotein cholesterol; LDL‐C, low‐density lipoprotein cholesterol; NCCL, nonculprit coronary lesion; non‐HDL‐C, nonhigh‐density lipoprotein cholesterol; TC, total cholesterol; TG, triglyceride.

**Figure 1:**
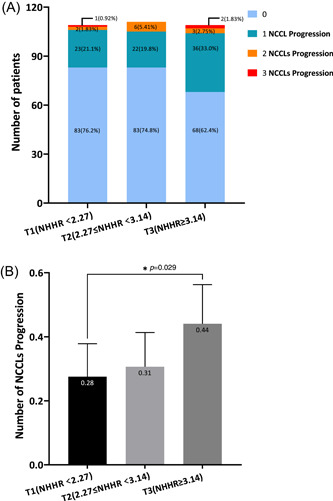
Number of vessels associated with NCCL progression among tertile groups of non‐HDL‐C/HDL‐C ratio. (A) The distribution of patients with the corresponding number of vessels associated with NCCL progression, presented as counts and percentages. (B) The mean value of the number of vessels associated with NCCL progression, with 95% confidence intervals. HDL‐C, high‐density lipoprotein cholesterol; NCCL, nonculprit coronary lesion; NHHR, non‐HDL‐C/HDL‐C ratio; non‐HDL‐C, nonhigh‐density lipoprotein cholesterol; T1, tertile 1; T2, tertile 2; T3, tertile 3.

In Table 3, the patients were divided into three groups based on the tertiles of the baseline non‐HDL‐C/HDL‐C ratio (tertile 1: *n* = 109, NHHR < 2.27; tertile 2: *n* = 111, 2.27 ≤ NHHR < 3.14; and tertile 3: *n* = 109 NHHR ≥ 3.14). Significant differences were observed across tertile groups of non‐HDL‐C/HDL‐C in terms of age, BMI, and previous PCI history. It was noted that individuals with higher tertile of non‐HDL‐C/HDL‐C tended to be younger (*p* = 0.003), have a higher BMI (*p* < 0.001), and were less likely to have a prior history of PCI (*p* < 0.001). Importantly, there were clear distinctions between the three groups in terms of the primary outcome measure, NCCL progression (*p* = 0.047), while, there was no significant difference in the median follow‐up time across these three groups (*p* = 0.84). Additionally, the number of vessels associated with NCCL progression at tertile 3 of non‐HDL‐C/HDL‐C was greater compared to that observed at tertile 1, and the differences were statistically significant (*p* = 0.029, Figure 1), but there were no significant differences in NCCL progression among tertile groups of LDL‐C despite a trend toward positive association (*p* = 0.37, Supporting Information: Figure 2).

**Table 3:** Characteristics and outcomes of the study population across tertile groups of the baseline non‐HDL‐C/HDL‐C ratio.

Variable	T1 (*n* = 109)	T2 (*n* = 111)	T3 (*n* = 109)	*p*
	NHHR < 2.27	2.27 ≤ NHHR < 3.14	NHHR ≥ 3.14	
Non‐HDL‐C/HDL‐C ratio	1.88 (1.54–2.10)	2.70 (2.51–2.90)	3.83 (3.44–4.35)	<0.001
Age (years)	64.6 ± 10.2	61.47 ± 11.11	59.73 ± 9.97	0.003
Male, *n* (%)	85 (77.9)	88 (79.3)	86 (70.9)	0.97
BMI (kg/m^2^)	24.66 ± 3.48	25.65 ± 3.01	26.34 ± 2.87	<0.001
SBP (mmHg)	134.5 ± 18.5	135.3 ± 21.2	138.4 ± 19.3	0.30
DBP (mmHg)	77.0 ± 12.6	81.16 ± 14.02	80.61 ± 11.33	0.03
Heart rate (beats/min)	75.6 ± 13.4	75.0 ± 10.7	76.3 ± 13.2	0.76
Hypertension, *n* (%)	80 (73.4)	77 (69.4)	72 (66.1)	0.499
Diabetes mellitus, *n* (%)	48 (44.0)	49 (44.1)	55 (50.5)	0.55
Smoking, *n* (%)	55 (50.5)	68 (61.3)	70 (64.2)	0.094
Previous MI, *n* (%)	22 (20.2)	15 (13.5)	12 (11.0)	0.14
Previous PCI, *n* (%)	57 (52.3)	44 (39.6)	23 (21.1)	<0.001
AMI, *n* (%)	19 (17.4)	15 (13.5)	26 (23.9)	0.13
No. of PCI‐treated lesion, *n*	1.72 ± 0.86	1.93 ± 1.05	1.62 ± 0.79	0.042
Lipid lowering drugs, *n* (%)	109 (100.0)	110 (99.1)	109 (100.0)	1
Antiplatelet drugs, *n* (%)	109 (100.0)	111 (100.0)	109 (100.0)	1
ACEI/ARB, *n* (%)	65 (59.6)	51 (46.0)	59 (54.1)	0.12
β‐blocker, *n* (%)	89 (81.7)	83 (74.8)	75 (68.8)	0.09
CCB, *n* (%)	32 (29.4)	40 (36.0)	26 (23.9)	0.14
Follow‐up period (years)	1.04 (0.80–1.79)	1.06 (0.74–1.97)	1.13 (0.85–1.94)	0.84
Baseline lipid parameter				
TC (mmol/L)	3.29 (2.86–3.81)	4.26 (3.58–4.62)	5.14 (4.31–5.73)	<0.001
TG (mmol/L)	1.17 (0.88–1.50)	1.58 (1.26–2.09)	2.26 (1.50–2.92)	<0.001
LDL‐C (mmol/L)	1.63 (1.43–1.94)	2.37 (2.05–2.76)	3.13 (2.66–3.65)	<0.001
Non‐HDL‐C (mmol/L)	2.05 (1.80–2.50)	3.08 (2.63–3.42)	4.07 (3.38–4.61)	<0.001
HDL‐C (mmol/L)	1.20 (1.01–1.43)	1.12 (0.96–1.26)	1.03 (0.90–1.17)	<0.001
Follow‐up lipid parameter				
TC (mmol/L)	3.18 (2.84–3.82)	3.43 (3.15–3.97)	3.54 (3.08–4.17)	0.017
TG (mmol/L)	1.15 (0.90–1.55)	1.38 (1.04–1.88)	1.46 (1.09–2.03)	<0.001
LDL‐C (mmol/L)	1.56 (1.30–1.97)	1.88 (1.62–2.25)	1.95 (1.58–2.38)	<0.001
Non‐HDL‐C (mmol/L)	1.99 (1.67–2.56)	2.29 (2.02–2.85)	2.49 (2.04–3.17)	<0.001
HDL‐C (mmol/L)	1.17 (1.02–1.37)	1.10 (0.96–1.21)	0.99 (0.88–1.11)	<0.001
Non‐HDL‐C/HDL‐C ratio	1.76 (1.41–2.13)	2.26 (1.86–2.64)	2.54 (2.02–3.09)	<0.001
QCA				
Baseline cumulative NCCL lengths (mm)	5.9 (0.0–15.2)	5.0 (0.0–14.8)	0.0 (0.0–15.6)	0.53
Follow‐up cumulative NCCL lengths (mm)	13.3 (0.0–25.6)	12.6 (0.0–25.3)	11.6 (2.4–26.4)	0.93
Change of cumulative lesion lengths (mm)	0.0 (0.0–10.0)	0.59 (0.0–9.9)	3.8 (0.0–13.0)	0.25
Pre‐existing stenosis (%)	26.9 (0.0–36.4)	0.0 (0.0–38.8)	0.0 (0.0–35.9)	0.32
Follow‐up stenosis (%)	40.3 (32.2–53. 5)	41.3 (32.9–64.3)	43.4 (32.2–56.2)	0.58
Max progression rate (%)	0.0 (0.0–20.1)	2.4 (0.0–29.7)	17.7 (0.0–39.8)	0.005
Outcomes				
ISR, *n* (%)	26 (23.9)	21 (18.9)	13 (11.9)	0.072
NCCL revascularization, *n* (%)	7 (6.42)	11 (9.91)	14 (12.84)	0.28
NCCL‐related AMI, *n* (%)	0 (0.00)	2 (1.80)	0 (0.00)	0.33
All AMI, *n* (%)	7 (6.42)	5 (4.50)	9 (8.26)	0.52
NCCL progression, *n* (%)	26 (23.9)	28 (25.2)	41 (37.6)	0.047

*Note*: Values are mean ± SD, median (interquartile range), or *n* (%).

Abbreviations: ACEI, angiotensin‐converting enzyme inhibitor; AMI, acute myocardial infarction; ARB, angiotensin II receptor blocker; BMI, body mass index; CCB, calcium channel blocker; DBP, diastolic blood pressure; HDL‐C, high‐density lipoprotein cholesterol; ISR, in‐stent restenosis; LDL‐C, low‐density lipoprotein cholesterol; MI, myocardial infarction; NCCL, nonculprit coronary lesion; NHHR, non‐HDL‐C/HDL‐C ratio; non‐HDL‐C, nonhigh‐density lipoprotein cholesterol; PCI, percutaneous coronary intervention; QCA, quantitative coronary angiography; SBP, systolic blood pressure; T1, tertile 1; T2, tertile 2; T3, tertile 3; TC, total cholesterol; TG, triglyceride.

An in‐depth analysis was conducted to investigate the factors associated with the risk of NCCL progression or revascularization. In the univariate analysis (Supporting Information: Table 1), non‐HDL‐C (*p* = 0.046) and non‐HDL‐C/HDL‐C (*p* = 0.007) were found to be associated with NCCL progression. Similarly, HDL‐C (*p* = 0.004) and non‐HDL‐C/HDL‐C (*p* = 0.014) were associated with NCCL revascularization according to the univariate analysis. In the multivariate analysis adjusting for age, sex, and BMI (Model 2), further adjusting for smoking, hypertension, and diabetes (Model 3), and further adjusting for previous PCI and time interval between the two CAGs (Model 4) (Table 4), there was no significant association between LDL‐C levels and NCCL progression or NCCL revascularization (*p* > 0.05 for all odds ratios [ORs]). However, higher non‐HDL‐C levels were significantly associated with NCCL progression in models 1, 2, and 4 (*p* < 0.05 for all ORs) while higher HDL‐C levels were consistently associated with a lower risk of NCCL progression and NCCL revascularization (*p* < 0.05 for all ORs). Additionally, a higher non‐HDL‐C/HDL‐C ratio was significantly associated with an increased risk of NCCL progression and revascularization. These associations remained consistent across adjusted models, suggesting the independent predictive value of this ratio for NCCL progression and revascularization.

**Table 4:** Multivariate logistic regression analysis of the risk factors for NCCL progression and revascularization.

*N* = 329	NCCL progression		NCCL revascularization	
	OR (95% CI)	*p*	OR (95% CI)	*p*
LDL‐C (mmol/L)				
Model 1	1.22 (0.92–1.63)	0.16	1.05 (0.68–1.62)	0.83
Model 2	1.27 (0.95–1.70)	0.10	1.00 (0.64–1.57)	0.98
Model 3	1.30 (0.97–1.75)	0.079	1.02 (0.65–1.60)	0.93
Model 4	1.23 (0.90–1.69)	0.20	0.95 (0.58–1.54)	0.83
Non‐HDL‐C (mmol/L)				
Model 1	1.26 (1.01–1.57)	0.046	1.01 (0.72–1.43)	0.94
Model 2	1.30 (1.03–1.63)	0.025	0.98 (0.69–1.40)	0.93
Model 3	1.33 (1.05–1.68)	0.016	1.00 (0.70–1.43)	0.99
Model 4	1.29 (1.01–1.65)	0.047	0.94 (0.64–1.38)	0.75
HDL‐C (mmol/L)				
Model 1	0.41 (0.16–1.05)	0.063	0.08 (0.01–0.45)	0.004
Model 2	0.40 (0.15–1.07)	0.067	0.07 (0.01–0.44)	0.005
Model 3	0.36 (0.13–1.01)	0.053	0.06 (0.01–0.39)	0.003
Model 4	0.31 (0.11–0.88)	0.028	0.05 (0.01–0.35)	0.002
Non‐HDL‐C/HDL‐C				
Model 1	1.36 (1.09–1.69)	0.007	1.41 (1.07–1.87)	0.014
Model 2	1.41 (1.12–1.78)	0.004	1.41 (1.06–1.88)	0.018
Model 3	1.47 (1.16–1.87)	0.002	1.47 (1.08–1.98)	0.013
Model 4	1.45 (1.14–1.86)	0.003	1.46 (1.07–1.98)	0.017

*Note*: Model 1: unadjusted for covariates. Model 2: adjusted for age, sex, and BMI. Model 3: adjusted for age, sex, BMI, smoking, hypertension, and diabetes mellitus. Model 4: adjusted for age, sex, BMI, smoking, hypertension, diabetes mellitus, previous PCI, and time interval between the two CAGs.

Abbreviations: BMI, body mass index; CAG, consecutive coronary angiography; CI, confidence interval; HDL‐C, high‐density lipoprotein cholesterol; LDL‐C, low‐density lipoprotein cholesterol; NCCL, nonculprit coronary lesion; non‐HDL‐C, nonhigh‐density lipoprotein cholesterol; OR, odds ratio; PCI, percutaneous coronary intervention.

## 4 DISCUSSION

Dyslipidemia is widely recognized as an independent risk factor for ASCVD. In patients with coronary heart disease, NCCL progression plays a significant role in clinical adverse events.^2^ To investigate the association between baseline routine lipid profiles and the progression of NCCL, our study analyzed data from 329 patients with ACS following PCI procedure. Our findings revealed a significant association between the ratio of non‐HDL‐C to HDL‐C and the progression of NCCL. Specifically, we observed that the group with NCCL progression had a higher average non‐HDL‐C/HDL‐C ratio. Moreover, when we divided the non‐HDL‐C/HDL‐C ratio into tertiles and compared the progression rates of NCCLs at different levels, we found that higher levels of non‐HDL‐C/HDL‐C were associated with increased rates of progression in NCCL. We chose to use logistic regression instead of Cox regression models because the follow‐up time was not driven by the “event” of interest, NCCL progression. In a significant portion of patients, the follow‐up time was scheduled at 1 year after the first PCI or was based on symptoms that were not necessarily related to cardiac issues, which did not indicate the timing of the first occurrence of NCCL progression. Therefore, we believed that the prerequisites for using Cox regression models were not met. Instead, we utilized logistic regression with adjustment for the follow‐up interval to minimize the impact of time. After adjusting for covariates such as age, sex, BMI, smoking, history of hypertension, diabetes, previous PCI, and time interval between the two CAGs, non‐HDL‐C/HDL‐C remained an independent risk factor for the progression of NCCL.

Lowering LDL‐C levels has been shown to have a positive impact on atherosclerosis progression, promoting plaque regression and stabilization, and ultimately reducing the occurrence of MACE.^17^, ^18^, ^19^, ^20^ However, in the present study, we did not find a significant association between LDL‐C levels and the progression of NCCL, although there was a trend suggesting a positive association. This might be due to the effective overall control of LDL‐C levels with widespread use of statins and other LDL‐C lowering drugs, which could blunt the association. Although this association may become evident with a larger sample size and longer follow‐up period, our findings indicated that non‐HDL‐C/HDL‐C serve as a stronger and earlier predictor for NCCL progression under current guideline‐directed lipid‐lowering therapy.

Recently, there has been a growing interest in the role of other atherogenic lipoproteins in the development of atherosclerosis and its associated clinical outcomes.^21^, ^22^, ^23^ A study involving 27 436 urban workers in China found a significant association between the non‐HDL‐C/HDL‐C ratio and the stability of carotid plaque.^24^ Likewise, a study of 426 patients with first‐onset non‐ST‐segment elevation acute myocardial infarction (NSTEMI) indicated that the non‐HDL‐C/HDL‐C ratio is significantly associated with severe coronary lesions and functions as an independent predictor of cardiovascular outcomes among NSTEMI patients.^25^ Furthermore, an analysis of 46 786 patients with type 2 diabetes showed that lower levels of non‐HDL‐C/HDL‐C are more effective risk markers for ASCVD compared to LDL‐C levels below 3 mmol/L.^26^ In a primary prevention study conducted on 220 postmenopausal women, the non‐HDL‐C/HDL‐C ratio showed a stronger association with increased carotid intima‐media thickness in comparison to other lipid parameters.^27^ Additionally, a study on 5822 Chinese individuals diagnosed with metabolic syndrome found a progressive increase in carotid intima‐media thickness across quartiles of the non‐HDL‐C/HDL‐C ratio, with no significant differences observed between the apoB/apoA1 ratio and non‐HDL‐C/HDL‐C ratio in predicting carotid atherosclerosis.^28^

Lipoproteins containing apoB have a significant impact on the development and progression of atherosclerotic plaques by accumulating in the arterial intima, leading to atherogenic effects.^29^ Non‐HDL‐C, which is calculated by subtracting HDL‐C from TC, provides an estimate of the levels of lipoproteins that contain apoB.^30^ Additionally, HDL‐C plays a crucial role in removing excess cholesterol from peripheral tissues and transporting it back to the liver.^31^ Therefore, the non‐HDL‐C/HDL‐C ratio represents cholesterol transport balance.

Plasma HDL levels and the incidence of ASCVD show a significant inverse correlation, as indicated by epidemiological studies.^32^ However, recent genetic studies and clinical trials investigating novel HDL‐raising agents have yielded contrasting results, failing to establish a definitive link between increased HDL levels and reduced heart disease risk.^33^, ^34^, ^35^ These findings highlight the inadequacy of our current understanding regarding HDL biology.

Furthermore, our study revealed that among ACS patients, those in the group with a higher non‐HDL‐C/HDL‐C ratio were younger and had a higher BMI. These observations suggest a possible higher prevalence of metabolic syndrome in these individuals. These findings align with previous research that suggests non‐HDL‐C/HDL‐C as a potential marker for metabolic risk and insulin resistance.^13^

The limitations of our study are as follows. First, it was a retrospective, single‐center cohort study, which may introduce selection bias. To validate our findings, larger‐scale prospective studies are necessary. Additionally, two‐thirds of the patients in our study did not have measurements for apoB and apoA1, as these indicators are not routinely measured. Further investigation can be conducted to explore the relationship between these indicators, their ratio, and changes in coronary artery lesions if a sufficient sample size is available. Moreover, the progression of coronary lesions may also be influenced by the nature, composition and load of plaques, but we lack intraluminal imaging data to delve into this phenomenon. Another limitation is the absence of inflammatory markers, such as hypersensitive C‐reactive protein, which has been reported in previous studies to be related to the progression of coronary artery disease.^15^ Although we excluded patients with acute infection to minimize confounding factors, an imbalance in chronic inflammatory status may still have an impact. It is important to note that the majority of participants were male, but there was no significant difference in sex composition between the different groups. Furthermore, blood lipids were measured randomly, as some patients with coronary heart disease had their blood drawn immediately upon admission instead of after fasting. However, previous studies have shown that random blood lipids can better reflect blood lipid load levels than fasting blood lipids.^36^, ^37^, ^38^, ^39^ In our study, LDL‐C was measured using the direct method, which is less affected by TG compared to Friedewald‐estimated levels and thus eliminated the need for fasting.^40^ TC and HDL‐C (and therefore non‐HDL‐C calculation values) levels also appear to be less affected by recent food intake, so fasting may not always be required. Last, our analysis was conducted on a group of Chinese patients, so further confirmational studies are needed to determine if our findings can be generalized to other racial groups.

## 5 CONCLUSIONS

In conclusion, our findings demonstrated that the ratio of non‐HDL‐C to HDL‐C can independently predict NCCL progression and NCCL revascularization in ACS patients after PCI. This ratio is easy to calculate and cost‐effective, making it a useful tool for assessing risk and improving ASCVD secondary prevention. Further research is necessary to validate these findings, gain a deeper understanding of the underlying mechanisms, and explore potential new targets for better reducing ASCVD residual risk.

## AUTHOR CONTRIBUTIONS

**Jiamin Liu**: Conceptualization; methodology; investigation; project administration; data curation; formal analysis; writing—original draft. **Li Zhao**: Conceptualization; methodology; formal analysis; writing—original draft. **Yazhou Zhang**: Conceptualization; methodology; data curation; writing—review & editing. **Lili Wang**: Methodology; investigation; data curation; writing—review & editing. **Qianqian Feng**: Methodology; investigation; data curation; writing—review & editing. **Jing Cui**: Methodology; investigation; writing—review & editing. **Wenhong Zhang**: Methodology; data curation; writing—review & editing. **Jianyong Zheng**: Conceptualization; methodology; writing—review & editing. **Dan Wang**: Data curation; writing—review & editing. **Fengjiao Zhao**: Data curation; writing—review & editing. **Jiangchun He**: Conceptualization; methodology; project administration; funding acquisition; writing—review & editing. **Yu Chen**: Conceptualization; methodology; project administration; writing—review & editing.

## CONFLICT OF INTEREST STATEMENT

The authors declare no conflict of interest.

## Supporting information

Supplemental Figure 1.

Supplemental Figure 2.

Supplementary Information

## Data Availability

The data that support the findings of this study are available from the corresponding author upon reasonable request.
