# Supplementary figures and images for: A higher non‐HDL‐C/HDL‐C ratio was associated with an increased risk of progression of nonculprit coronary lesion in patients with acute coronary syndrome undergoing percutaneous coronary intervention
Source: Clin Cardiol. 2024 Feb 25;47(2):e24243. doi: 10.1002/clc.24243 (PMC10894525; doi:10.1002/clc.24243)

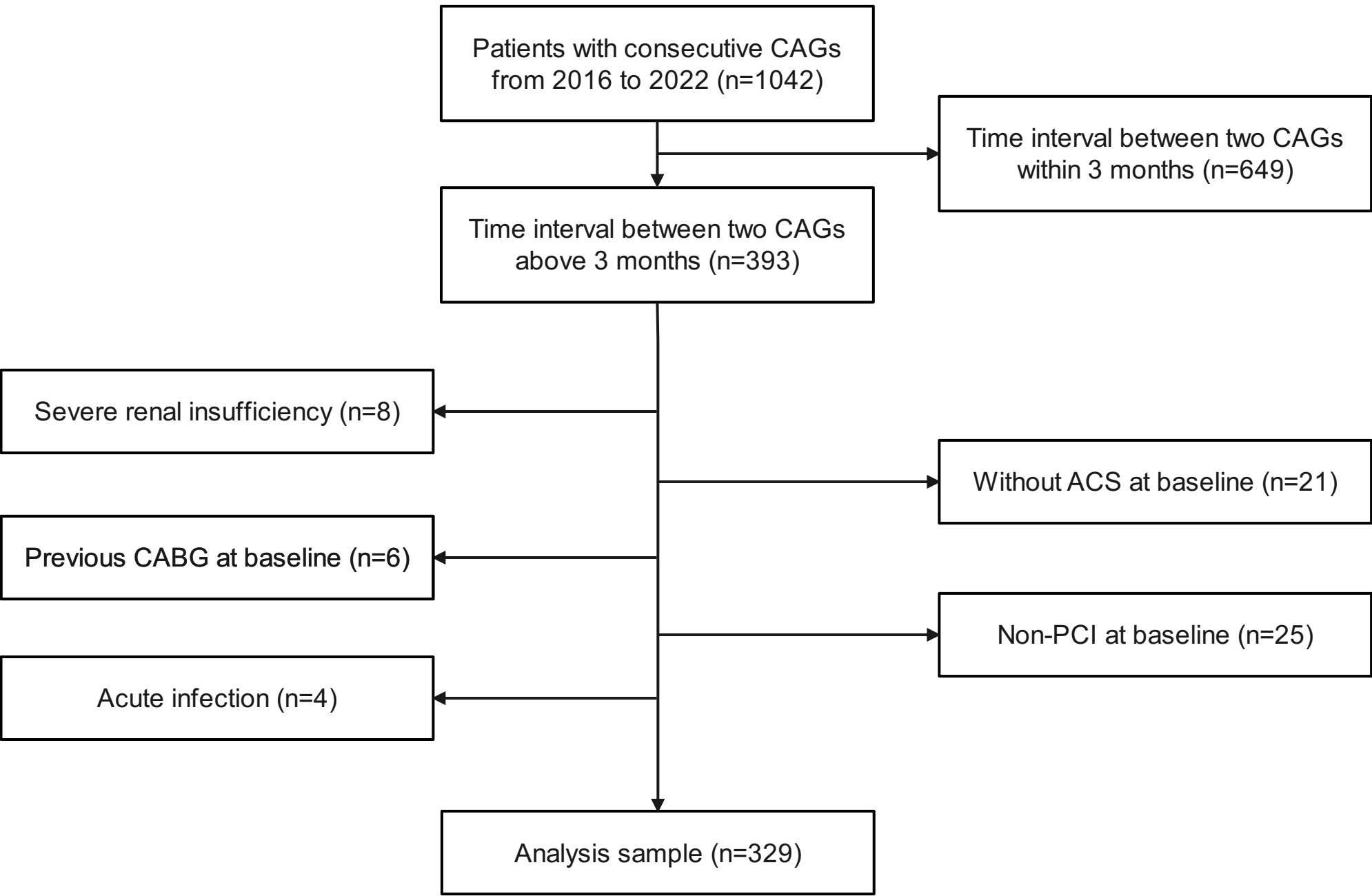

Supplemental Figure 1 Study Population Flowchart

Supplement: Supplementary file 1 — Supplemental Figure 1. [file CLC-47-e24243-s001.pdf]

**A**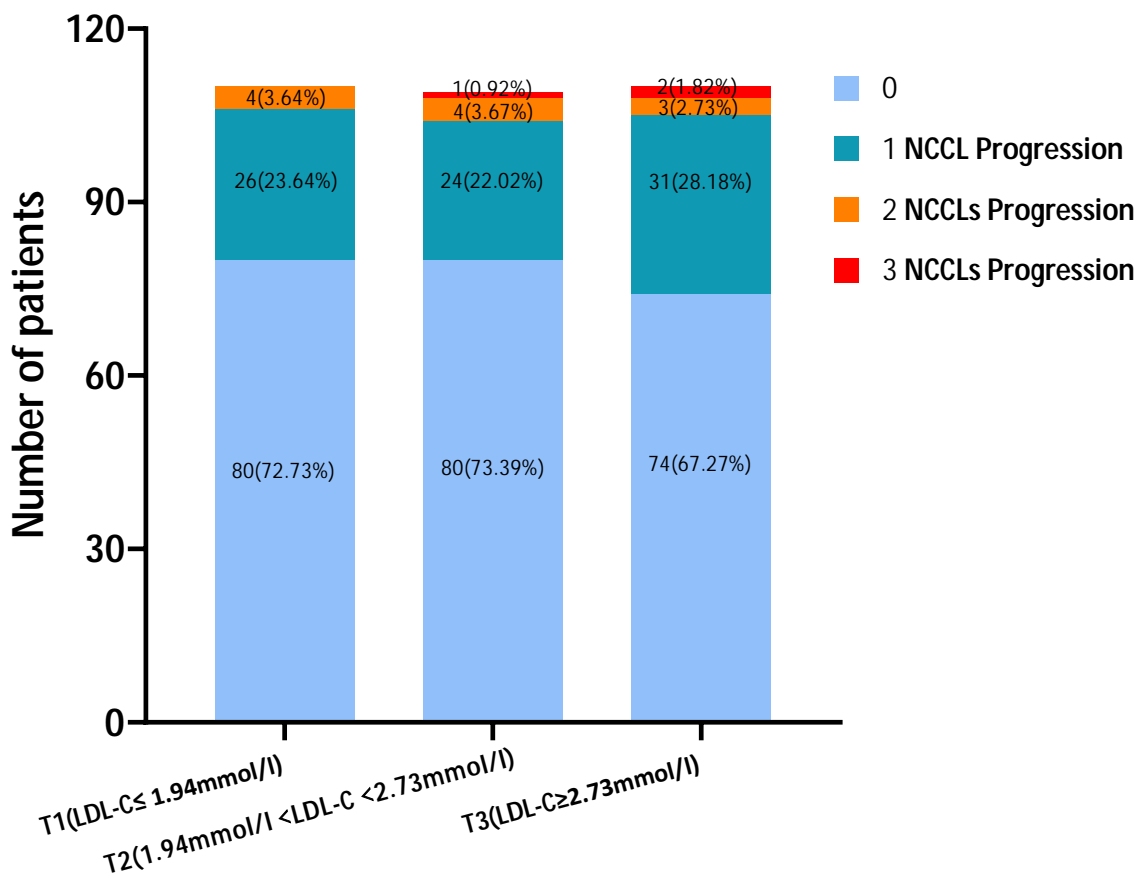**B**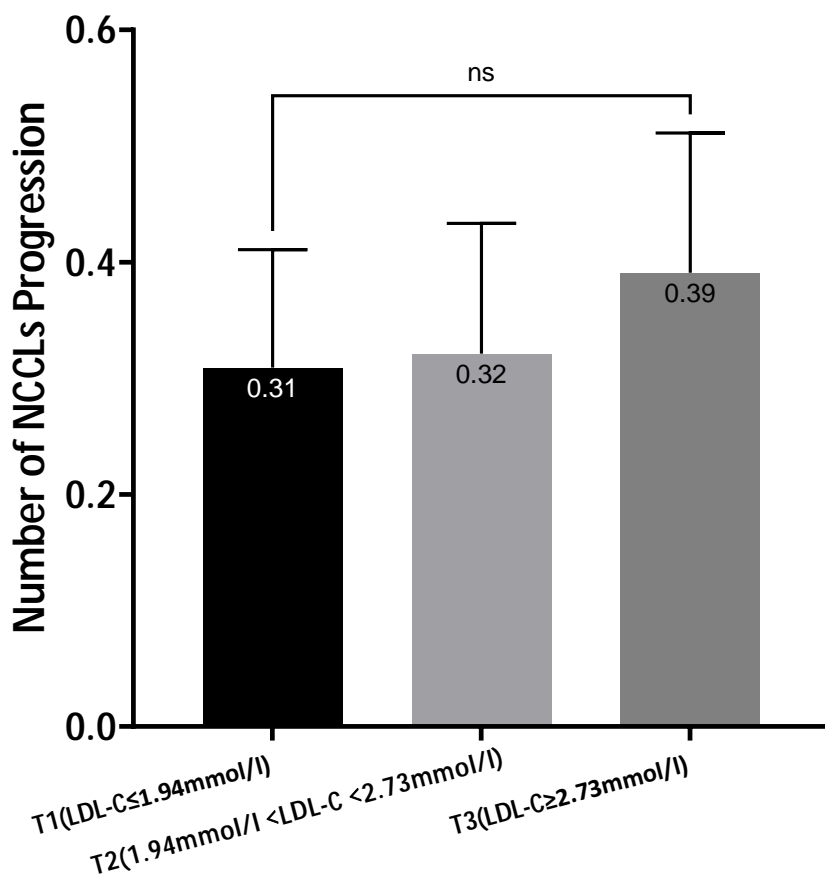

Supplement: Supplementary file 2 — Supplemental Figure 2. [file CLC-47-e24243-s003.pdf]
