# Supplementary material for: A higher non‐HDL‐C/HDL‐C ratio was associated with an increased risk of progression of nonculprit coronary lesion in patients with acute coronary syndrome undergoing percutaneous coronary intervention
Source: Clin Cardiol. 2024 Feb 25;47(2):e24243. doi: 10.1002/clc.24243 (PMC10894525; doi:10.1002/clc.24243)
Supplement: Supplementary file 3 — Supplementary Information [file CLC-47-e24243-s002.docx]

**Supplemental Table 1 Univariate logistic regression analysis of the risk factors for NCCL progression and revascularization.**

| **N=329** | **NCCL progression** | | | **NCCL revascularization** | |
| --- | --- | --- | --- | --- | --- |
|  | **OR (95%CI)** | **P** | **OR (95%CI)** | | **P** |
| Age, years | 1.01 (0.99 - 1.03) | 0.35 | 1.00 (0.96 - 1.03) | | 0.80 |
| BMI, kg/m^2 | 1.02 (0.95 - 1.10) | 0.62 | 1.09 (0.97 - 1.23) | | 0.14 |
| Male | 1.22 (0.67 - 2.22) | 0.51 | 0.79 (0.34 - 1.85) | | 0.59 |
| Hypertension | 1.32 (0.78 - 2.25) | 0.31 | 1.35 (0.58 - 3.11) | | 0.49 |
| Diabetes mellitus | 0.95 (0.59 - 1.53) | 0.83 | 0.90 (0.43 - 1.87) | | 0.77 |
| Smoking | 0.96 (0.59 - 1.55) | 0.86 | 0.90 (0.43 - 1.87) | | 0.77 |
| TC, mmol/L | 1.16 (0.94 - 1.42) | 0.16 | 0.90 (0.65 - 1.25) | | 0.54 |
| TG, mmol/L | 1.13 (0.95 - 1.35) | 0.16 | 0.93 (0.67 - 1.30) | | 0.68 |
| LDL-C, mmol/L | 1.22 (0.92 - 1.63) | 0.16 | 1.05 (0.68 - 1.62) | | 0.83 |
| non-HDL-C, mmol/L | 1.26 (1.01 - 1.57) | 0.046 | 1.01 (0.72 - 1.43) | | 0.94 |
| HDL-C, mmol/L | 0.41 (0.16 - 1.05) | 0.063 | 0.08 (0.01 - 0.45) | | 0.004 |
| non-HDL-C/HDL-C | 1.36 (1.09 - 1.69) | 0.007 | 1.41 (1.07 - 1.87) | | 0.014 |

NCCL: nonculprit coronary lesion; OR: odds ratio; CI: confidence interval; BMI: body mass index; TC: total cholesterol; TG: triglyceride; LDL-C: low-density lipoprotein cholesterol; non-HDL-C: non-high-density lipoprotein cholesterol; HDL-C: high-density lipoprotein cholesterol; non-HDL-C/HDL-C: non-high-density-lipoprotein-cholesterol/high-density-lipoprotein-cholesterol ratio.
